# LncRNA-PRLB drives ovarian cancer progression and chemoresistance by stabilizing GPX4 mRNA through the FUS-mediated suppression of ferroptosis

**DOI:** 10.3389/fmed.2026.1759058

**Published:** 2026-02-17

**Authors:** Li Jiang, Jie Pi, Na Li, Jing Cao, Yuzi Zhao

**Affiliations:** 1 Department of Obstetrics and Gynecology, Renmin Hospital of Wuhan University, Wuhan, China; 2 Department of Obstetrics and Gynecology, Jiayu County Maternal and Child Health Hospital, Jiayu, Hubei, China; 3 Department of Obstetrics and Gynecology, Wuhan Caidian District Maternal and Child Health Hospital of Wuhan, Wuhan, China

**Keywords:** chemoresistance, ferroptosis, FUS, GPX4, lncRNA-PRLB, ovarian cancer

## Abstract

**Background:**

Ovarian cancer is highly lethal, largely due to the rapid development of paclitaxel resistance. This study aimed to determine whether progression-associated lncRNA in breast cancer (lncRNA-PRLB) regulates ferroptosis and paclitaxel resistance in ovarian cancer and to elucidate the underlying mechanism.

**Methods:**

Functional assays, including 5-ethynyl-2′-deoxyuridine (EdU) incorporation, Cell Counting Kit-8 (CCK-8) viability measurements, terminal deoxynucleotidyl transferase dUTP nick end labeling (TUNEL) staining, caspase-3 activity assays, Transwell invasion, wound healing, and ferroptosis marker analyses [reactive oxygen species (ROS), malondialdehyde MDA, Fe^2+^, lactate dehydrogenase (LDH), and glutathione (GSH)], were performed in CAOV3 and SKOV3 ovarian cancer cells following lncRNA-PRLB knockdown or overexpression. RNA pull-down, RNA immunoprecipitation (RIP), and actinomycin D mRNA decay assays were conducted to elucidate the molecular interactions between lncRNA-PRLB, the RNA-binding protein fused in sarcoma (FUS), and glutathione peroxidase 4 (GPX4) mRNA. Rescue experiments involving GPX4 overexpression or ferrostatin-1 treatment, as well as FUS knockdown, were used to define mechanistic dependencies. Paclitaxel sensitivity assays were performed to assess chemoresistance phenotypes.

**Results:**

LncRNA-PRLB was identified as an oncogenic regulator that enhances ovarian cancer cell proliferation, migration, invasion, and paclitaxel resistance. Silencing lncRNA-PRLB induced apoptosis and triggered ferroptosis, characterized by elevated ROS, MDA, Fe^2+^, LDH release, and depletion of GSH. Mechanistically, lncRNA-PRLB directly bound to FUS and facilitated the stabilization of GPX4 mRNA, thereby maintaining GPX4 protein levels and suppressing ferroptotic signaling. GPX4 overexpression or ferroptosis inhibition rescued the phenotypes induced by lncRNA-PRLB knockdown, while FUS depletion abolished the oncogenic functions of lncRNA-PRLB. Loss of the lncRNA-PRLB/FUS/GPX4 axis sensitized ovarian cancer cells to paclitaxel, suggesting that ferroptosis suppression is a key driver of chemoresistance.

**Conclusion:**

This study identifies lncRNA-PRLB as a critical upstream regulator of ferroptosis resistance and chemoresistance in ovarian cancer. By scaffolding FUS to stabilize GPX4 mRNA, lncRNA-PRLB maintains GPX4 expression and enables tumor cells to evade ferroptotic cell death.

## Introduction

Ovarian cancer is the most lethal gynecologic malignancy, with over 300,000 new cases and more than 200,000 deaths reported annually worldwide (1, 2). The high mortality rate is mainly attributed to late-stage diagnosis, extensive intraperitoneal dissemination, and the rapid emergence of chemoresistance (3, 4). Although cytoreductive surgery combined with platinum- and taxane-based chemotherapy remains the standard intervention, the majority of patients experience relapse within 2 years, ultimately developing resistance to frontline drugs such as paclitaxel (5–7). The mechanisms that drive ovarian cancer progression and chemoresistance are complex and multifactorial, involving genetic alterations, metabolic rewiring, and dysregulated cell death pathways (8, 9). Thus, discovering novel molecular regulators that contribute to ovarian tumor growth and drug response is critical for improving patient outcomes.

Long non-coding RNAs (lncRNAs) have emerged as key regulators of cancer biology (10). LncRNAs exert diverse functions by interacting with DNA, RNA, or proteins to modulate chromatin structure, transcriptional activation or suppression, mRNA stability, and protein translation (11). Accumulating evidence has shown that lncRNAs can act as oncogenes or tumor suppressors and are closely related to proliferation, metastasis, immune evasion, metabolic plasticity, and chemotherapy resistance (12). Despite expanding interest in lncRNA-mediated regulation, many lncRNAs remain unexplored, and their contributions to ovarian cancer pathogenesis remain poorly defined. Previous studies have identified lncRNA-PRLB (progression-associated lncRNA in breast cancer) as a previously uncharacterized transcript that is highly expressed in ovarian cancer cells, implicating it as a potential driver of tumor progression and therapeutic resistance (13). Consistent with this notion, our previous study revealed that lncRNA-PRLB promotes paclitaxel resistance in ovarian cancer by activating the remodeling and spacing factor 1/nuclear factor kappa B (RSF1/NF-κB) signaling pathway (14). However, detailed underlying molecular mechanisms of lncRNA-PRLB in ovarian cancer progression remain elusive.

One mechanism by which lncRNAs exert their biological effects is through interactions with RNA-binding proteins (RBPs) (11). RBPs such as fused in sarcoma (FUS) are known to regulate transcript stability, alternative splicing, transport, and translation (15). Dysregulation of RBP–RNA interactions has been increasingly recognized as a key driver of oncogenic signaling (16). FUS, in particular, has been implicated in multiple cancer-related processes and functions as a modulator of mRNA stabilization (17–19). However, its involvement in ovarian cancer biology, especially in the context of lncRNA-mediated regulation of cell death pathways, has not been fully elucidated. Whether specific lncRNAs recruit FUS to stabilize oncogenic transcripts in ovarian cancer remains an important unanswered question.

Ferroptosis, a regulated form of cell death characterized by iron accumulation, lipid peroxidation, and excessive oxidative stress, has recently gained considerable attention in the field of cancer biology (20). Unlike apoptosis or necrosis, ferroptosis is uniquely driven by metabolic and redox imbalances, making it an attractive target for drug-resistant cancers (21). Glutathione peroxidase 4 (GPX4) is a major suppressor of ferroptosis, functioning to detoxify lipid hydroperoxides and maintain membrane integrity (22). Elevated GPX4 expression has been associated with ferroptosis resistance and chemotherapy tolerance across multiple cancer types (23). In ovarian cancer, ferroptosis resistance has been proposed as a contributor to platinum resistance (24, 25); however, the upstream mechanisms that control GPX4 expression and ferroptosis susceptibility remain poorly characterized. The possibility that lncRNAs may regulate GPX4 stability and ferroptosis responses through interaction with RBPs remains largely unexplored.

In light of these observations, we aimed to elucidate the molecular basis by which lncRNA-PRLB contributes to ovarian cancer progression and chemoresistance. Based on preliminary findings and the known role of ferroptosis in therapy response, we hypothesized that lncRNA-PRLB promotes ovarian cancer cell survival and paclitaxel resistance by suppressing ferroptosis through a post-transcriptional regulatory mechanism. Specifically, we posited that lncRNA-PRLB interacts with the RNA-binding protein FUS to stabilize GPX4 mRNA, a central inhibitor of ferroptotic cell death, thereby maintaining a ferroptosis-resistant state that supports tumor growth and drug tolerance. To test this hypothesis, we performed comprehensive functional, biochemical, and molecular assays to define the role of the lncRNA-PRLB/FUS/GPX4 axis in ovarian cancer biology. This study aimed to establish lncRNA-PRLB as a key upstream regulator of ferroptosis and chemoresistance and to identify a potential therapeutic target for overcoming paclitaxel resistance in ovarian cancer.

## Materials and methods

### Cell culture

Human ovarian cancer cell lines CAOV3 and SKOV3 were obtained from the American Type Culture Collection (ATCC, Manassas, USA). The cells were cultured in RPMI-1640 medium (Gibco, Waltham, USA) supplemented with 10% fetal bovine serum (FBS; Gibco) and 1% penicillin–streptomycin (Gibco). The Tax-resistant ovarian cancer cell lines SKOV3/Tax and CAOV3/Tax were generated according to our previous study (14). In brief, paclitaxel-resistant derivatives (CAOV3/Tax and SKOV3/Tax) were generated by stepwise exposure of parental CAOV3 and SKOV3 cells to increasing concentrations of paclitaxel (1–30 nM). Paclitaxel was increased by 2 nM every other week until 10 nM was reached. After cells were maintained at 10 nM for 2 months, the concentration was further increased by 5 nM monthly until 30 nM was achieved. The SKOV3/Tax and CAOV3/Tax cell lines were maintained in 30 nM Tax to keep the drug-resistant phenotype. The above-obtained drug-resistant cell lines were maintained in Tax-free medium for at least 2 weeks before the experiment.

### SiRNA and plasmid transfection

Chemically synthesized siRNAs targeting lncRNA-PRLB and FUS (GenePharma, Shanghai, China) were used at a final concentration of 50 nM. Scrambled siRNA (siNC; GenePharma) served as a negative control. The siRNA sequences are presented in Supplementary Table S1. Overexpression plasmids pcDNA3.1-lncRNA-PRLB and pcDNA3.1-GPX4 were purchased from GenePharma. An empty pcDNA3.1 vector (Invitrogen, Carlsbad, USA) served as a control. Transfections were performed using Lipofectamine 3000 (Invitrogen) diluted in Opti-MEM (Gibco). After 6 h, the medium was replaced, and the cells were incubated for 48 h before downstream analysis.

### Ferrostatin-1 treatment

Ferroptosis inhibition was achieved by treatment with ferrostatin-1 (Fer-1; MedChemExpress, New Jersey, USA, Cat#HY-100579). Fer-1 was dissolved in DMSO (Sigma, St. Louis, USA) to create a 10 mM stock solution. The cells were pretreated with 20 μM Fer-1 for 2 h prior to siRNA or plasmid transfection and maintained in Fer-1-containing medium for the duration of the assay.

### RNA extraction and quantitative real-time PCR (qRT-PCR)

Total RNA was extracted using TRIzol reagent (Invitrogen). RNA concentrations were measured using NanoDrop 2000 (Thermo Fisher Scientific, Waltham, USA). cDNA was synthesized using the PrimeScript RT Reagent Kit (Takara, Dalian, China). PCR amplification was performed using SYBR Premix Ex Taq II (Takara) on a QuantStudio 6 Flex Real-Time PCR system (Applied Biosystems). All primer sets were obtained from Sangon Biotech (Shanghai, China). Relative expression levels were calculated using the 2^*−ΔΔ*Ct^ method.

### Western blotting

The cells were lysed in radioimmunoprecipitation assay buffer (Beyotime, Beijing, China) supplemented with protease inhibitor cocktails (Roche). Protein concentrations were quantified using the BCA Protein Assay Kit (Thermo Fisher Scientific, Waltham, USA). The proteins were separated using 10% SDS-PAGE gels (Bio-Rad) and transferred to polyvinylidene fluoride membranes (Millipore, Waltham, USA, Cat#IPVH00010). The membranes were blocked with 5% skim milk and probed overnight at 4 °C with primary antibodies, including anti-GPX4 (1:1000; Abcam, Cambridge, UK), anti-FUS (1:1000; CST, Danvers, USA), and anti-GAPDH (1:3000; Proteintech, Rosemont, USA). Horseradish peroxidase-conjugated secondary antibodies (1:2000; CST) were applied for 1 h at room temperature. Signal detection was performed using enhanced chemiluminescence substrate (Thermo Fisher Scientific), and densitometric quantification was carried out using ImageJ.

### EdU incorporation assay

Cell proliferation was assessed using the EdU Apollo 567 Fluorescent Kit (RiboBio, Guangzhou, China). Cells seeded on glass coverslips were incubated with 50 μM EdU for 2 h, fixed with 4% paraformaldehyde (Solarbio, Beijing, China), and permeabilized with 0.5% Triton X-100 (Sigma, Cat#T8787). Fluorescence was visualized using an Olympus IX73 microscope.

### Cell Counting Kit-8 (CCK-8) assay

Cell viability was quantified using the CCK-8 kit (Dojindo, Tokyo, Japan). After the treatment, the cells were incubated with CCK-8 reagent for 2 h at 37 °C, and absorbance at 450 nm was measured using a SpectraMax M2 microplate reader (Molecular Devices, San Jose, USA). For IC₅₀ determination, the cells were exposed to serial dilutions of paclitaxel (Sigma, Cat#T7191) for 48 h.

### TUNEL staining

Apoptosis was assessed using the One-Step TUNEL Apoptosis Kit (Beyotime). After fixation and permeabilization, the cells were incubated with the TUNEL reaction mixture for 1 h at 37 °C. 4′,6-diamidino-2-phenylindole (DAPI) counterstaining was performed using the Fluoromount Aqueous Mounting Medium (Sigma). Images were analyzed using a confocal microscope.

### Caspase-3 activity assay

Caspase-3 activity was measured using a commercial caspase-3 activity assay kit (Beyotime). Lysates were incubated with Ac-DEVD-pNA substrate for 2 h at 37 °C, and absorbance at 405 nm was recorded.

### Transwell invasion assay

Transwell inserts with 8-μm pores (Corning, USA) were coated with Matrigel (BD Biosciences, San Jose, USA) diluted 1:8. After 24 h of incubation, invaded cells were fixed with 4% paraformaldehyde, stained with 0.1% crystal violet (Sigma), and counted under a light microscope.

### Wound healing assay

Cells with different treatments were allowed to grow into a monolayer. A uniform scratch was generated with a sterile 200-μL pipette tip. After washing with phosphate-buffered saline (PBS), the cells were maintained in serum-free RPMI-1640. Images were captured at 0 and 24 h using a light microscope, and migration distances were analyzed using ImageJ.

### Reactive oxygen species (ROS) detection

ROS levels were measured using the DCFH-DA probe (Beyotime). The cells were incubated with 10 μM DCFH-DA for 30 min at 37 °C. Fluorescence (Ex/Em: 488/525 nm) was quantified using either a microplate reader or a fluorescence microscope.

### Malondialdehyde (MDA), Fe^2+^, lactate dehydrogenase (LDH), and glutathione (GSH) assays

MDA levels were assessed using the Lipid Peroxidation MDA Assay Kit (Beyotime). Intracellular Fe^2+^ was quantified using an Iron Colorimetric Assay Kit (Abcam). LDH release was measured using the LDH Cytotoxicity Assay Kit (Beyotime). GSH levels were measured using the GSH/glutathione disulfide (GSSG) Detection Kit (Beyotime). Absorbance readings were normalized to total protein content.

### RNA pull-down assay

Biotin-labeled RNAs were synthesized using the Biotin RNA Labeling Kit (Thermo Fisher Scientific). CAOV3 lysates were incubated with biotinylated lncRNA-PRLB or GPX4 transcripts overnight, followed by capture using streptavidin magnetic beads (Invitrogen, Cat#11205D). After washing, the FUS protein was detected using Western blotting.

### RNA immunoprecipitation (RIP)

RIP assays were conducted using the Magna RIP Kit (Millipore). Lysates were immunoprecipitated with anti-FUS antibody (CST) or IgG (Millipore). Co-precipitated RNAs were purified and analyzed using quantitative real-time polymerase chain reaction (qRT-PCR).

### Actinomycin D mRNA stability assay

The cells were treated with 10 μg/mL actinomycin D (Sigma), and RNA samples were collected at 0, 2, 4, 6, and 8 h. GPX4 mRNA decay rates were calculated under conditions of lncRNA-PRLB knockdown, lncRNA-PRLB overexpression, or FUS knockdown.

### Statistical analysis

All experiments were performed in triplicate. Data are presented as mean ± standard deviation. Statistical significance was determined using two-tailed Student’s *t*-tests or one-way ANOVA with Tukey’s *post-hoc* test (GraphPad Prism 9). A *p*-value of < 0.05 was considered statistically significant.

## Results

### Effects of lncRNA-PRLB knockdown on ovarian cancer cell progression

**Figure 1:**
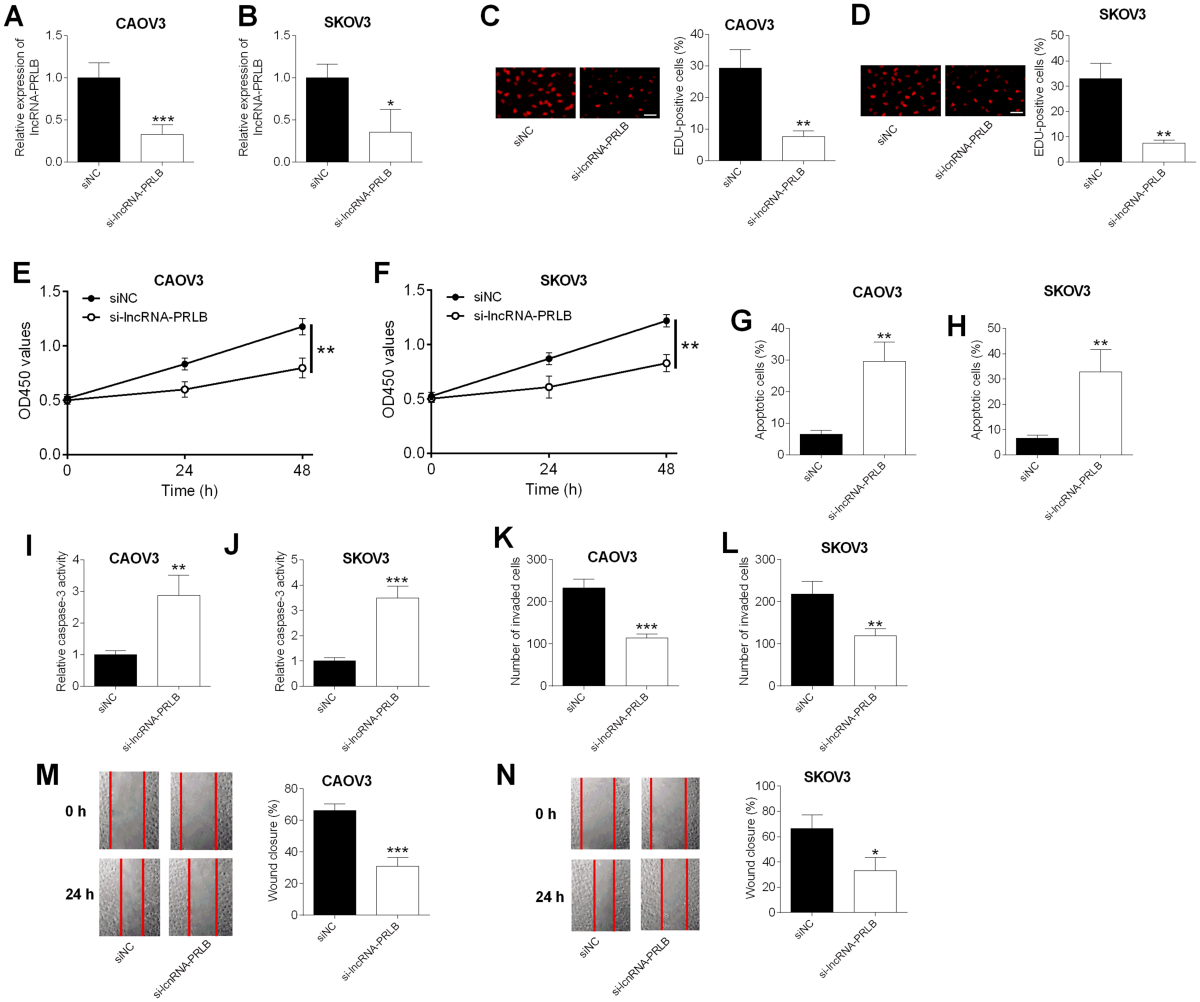
Effects of lncRNA-PRLB knockdown on ovarian cell progression and drug resistance. CAOV3 and SKOV3 cells were transfected with scrambled siRNA (siNC) or lncRNA-PRLB siRNA; 48 h after transfection, **(A,B)** lncRNA-PRLB expression was determined using qRT-PCR; **(C,D)** cell proliferation was evaluated using the EdU incorporation assay; **(E,F)** cell viability was determined using the CCK-8 assay; **(G,H)** cell apoptosis was measured using the TUNEL assay; **(I,J)** caspase-3 activity was determined using the caspase-3 activity assay; **(K,L)** cell invasion was assessed using the Transwell invasion assay; and **(M,N)** cell migration was determined using the wound healing assay. *N* = 3; *p < 0.05, **p < 0.01, and ***p < 0.001.

CAOV3 and SKOV3 cells were transfected with lncRNA-PRLB siRNA or scrambled siRNA (siNC). qRT-PCR analysis confirmed that lncRNA-PRLB was markedly reduced in CAOV3 and SKOV3 cells following lncRNA-PRLB siRNA transfection (Figures 1A,B).

Silencing lncRNA-PRLB significantly inhibited cell proliferation, as demonstrated by a reduction in EdU-positive cells in CAOV3 (Figure 1C) and SKOV3 (Figure 1D) cells. Consistently, CCK-8 assays revealed that the knockdown of lncRNA-PRLB markedly decreased cell viability in these cell lines (Figures 1E,F).

LncRNA-PRLB knockdown also increased apoptosis in ovarian cancer cells. TUNEL staining revealed an elevation of apoptotic cells in CAOV3 (Figure 1G) and SKOV3 (Figure 1H) following lncRNA-PRLB siRNA transfection. Further supporting this finding, caspase-3 activity was significantly enhanced in both CAOV3 (Figure 1I) and SKOV3 (Figure 1J) cells upon lncRNA-PRLB knockdown.

The impact of lncRNA-PRLB on metastatic behaviors was assessed using Transwell invasion and wound healing assays. Silencing lncRNA-PRLB decreased the invaded cell number in CAOV3 (Figure 1K) and SKOV3 (Figure 1L) cells. Similarly, wound healing results indicated that lncRNA-PRLB silencing markedly reduced the migratory capacity of CAOV3 (Figure 1M) and SKOV3 (Figure 1N) cells.

### Overexpression of lncRNA-PRLB promotes proliferation, viability, and metastatic behaviors while suppressing apoptosis in ovarian cancer cells

**Figure 2:**
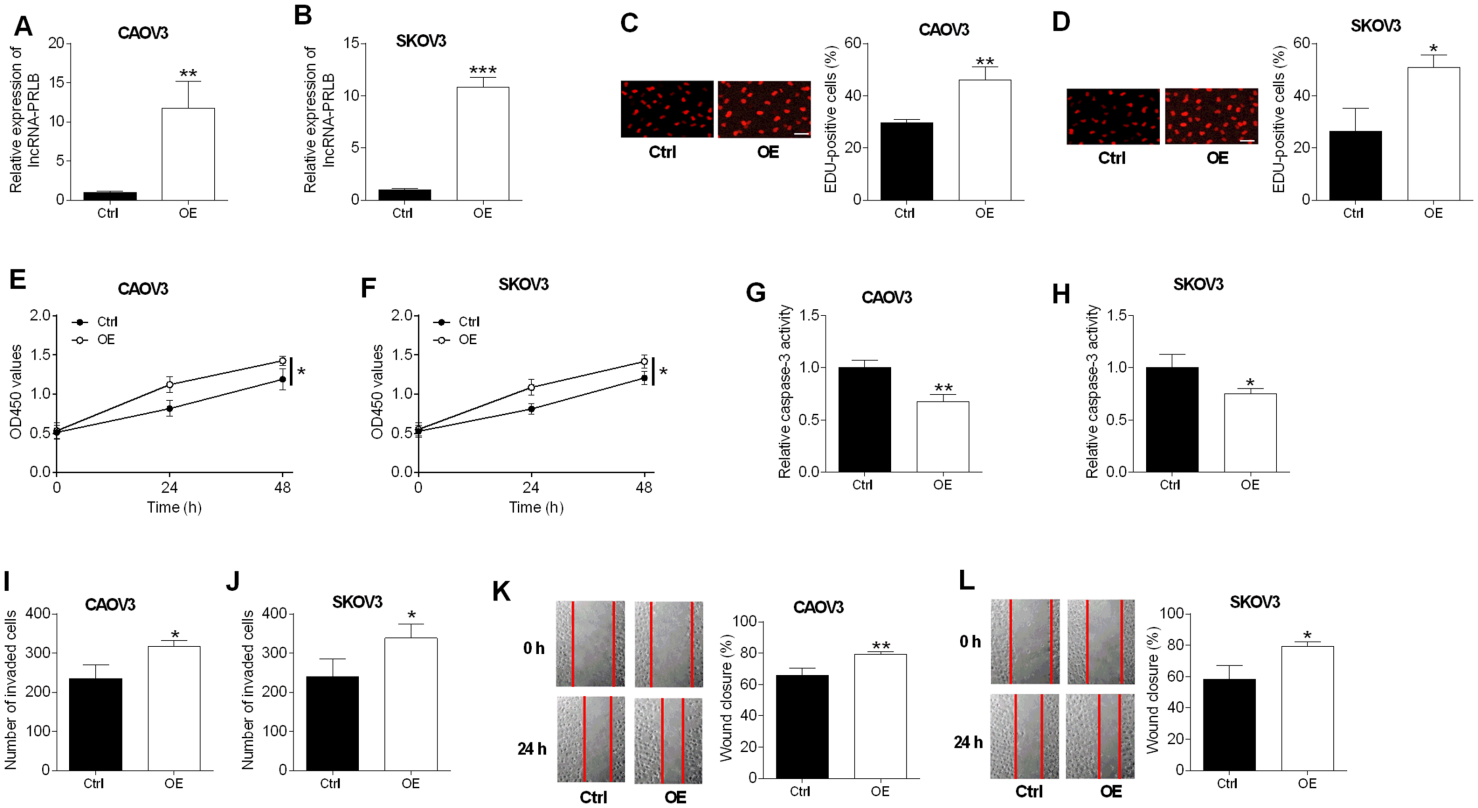
Effects of lncRNA-PRLB overexpression on ovarian cell progression and drug resistance. CAOV3 and SKOV3 cells were transfected with empty plasmid (Ctrl group) or lncRNA-PRLB-overexpressing vector (OE group); 48 h after transfection, **(A,B)** lncRNA-PRLB expression was determined using qRT-PCR; **(C,D)** cell proliferation was evaluated using the EdU incorporation assay; **(E,F)** cell viability was determined using the CCK-8 assay; **(G,H)** caspase-3 activity was determined using the caspase-3 activity assay; **(I,J)** cell invasion was assessed using the Transwell invasion assay; and **(K,L)** cell migration was determined using the wound healing assay. *N* = 3; *p < 0.05, **p < 0.01, and ***p < 0.001.

CAOV3 and SKOV3 cells were transfected with an lncRNA-PRLB-overexpressing plasmid or an empty vector. qRT-PCR confirmed that lncRNA-PRLB levels were elevated in both CAOV3 and SKOV3 cells following transfection with the overexpression construct (Figures 2A,B).

An enhanced expression of lncRNA-PRLB markedly promoted cell proliferation, as reflected by the increased number of EdU-positive cells in CAOV3 (Figure 2C) and SKOV3 (Figure 2D) cells. Consistent with this pro-proliferative effect, lncRNA-PRLB overexpression increased cell viability in both CAOV3 (Figure 2E) and SKOV3 (Figure 2F) cells.

In contrast to the knockdown experiments, the overexpression of lncRNA-PRLB reduced apoptosis-related activity. Caspase-3 activity was significantly decreased in CAOV3 (Figure 2G) and SKOV3 (Figure 2H) cells following lncRNA-PRLB overexpression, indicating the suppression of apoptotic signaling.

LncRNA-PRLB overexpression also enhanced metastatic cellular behaviors. Transwell assays revealed that the invaded cell number was increased in CAOV3 (Figure 2I) and SKOV3 (Figure 2J) cells. Moreover, wound healing assays revealed that lncRNA-PRLB overexpression significantly accelerated the migratory capacity of CAOV3 (Figure 2K) and SKOV3 (Figure 2L) cells.

### LncRNA-PRLB knockdown induces ferroptosis in ovarian cancer cells

**Figure 3:**
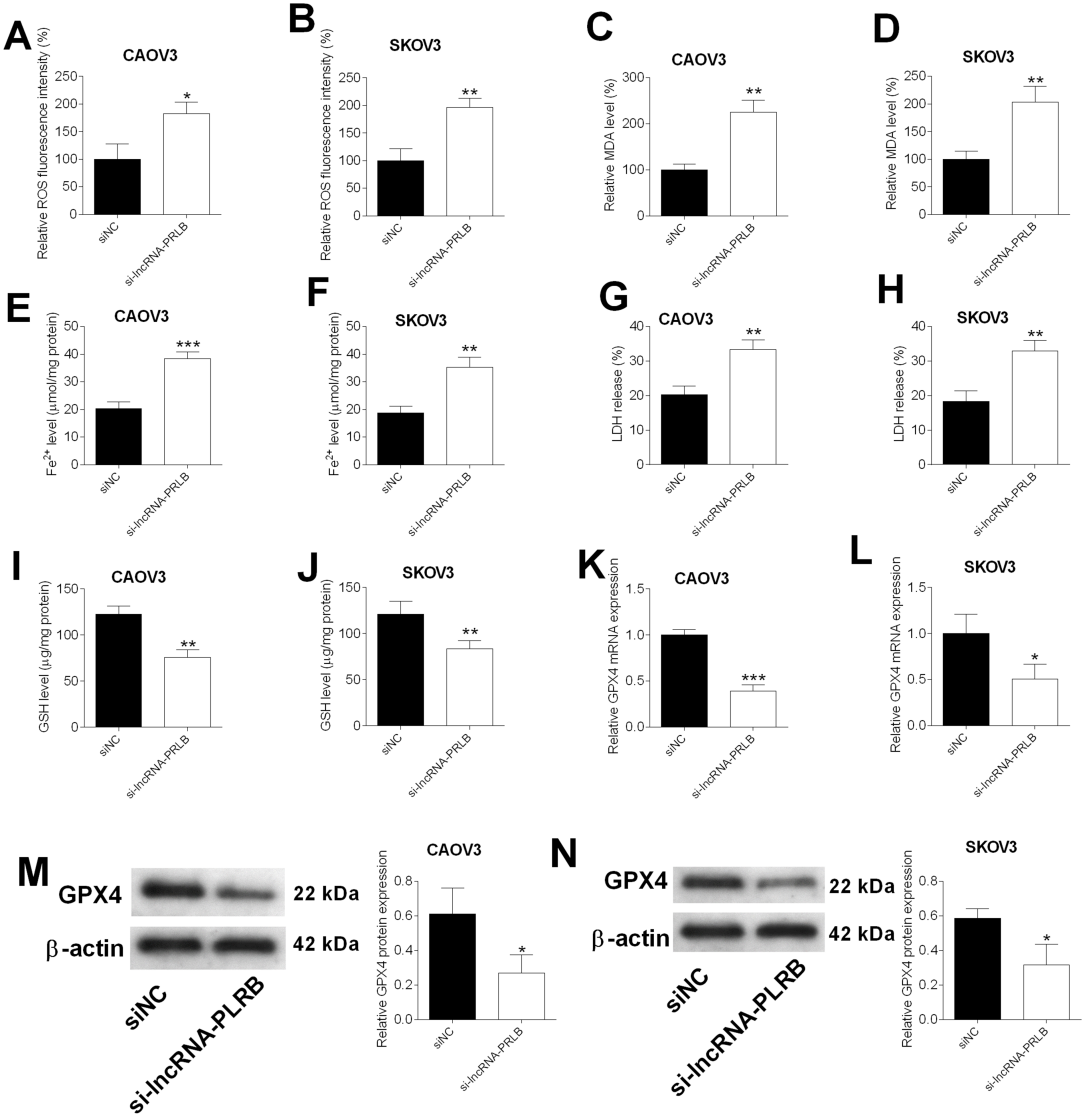
Effects of lncRNA-PRLB knockdown on the ferroptosis of ovarian cancer cells. CAOV3 and SKOV3 cells were transfected with scrambled siRNA (siNC) or lncRNA-PRLB siRNA; 48 h after transfection, **(A,B)** ROS levels were determined using the ROS fluorescence detection kit; **(C,D)** MDA levels were measured using the MDA kit; **(E,F)** Fe2 + levels in cells were measured using the Fe2 + content assay kit; **(G,H)** LDH levels were measured using the LDH release assay; **(I,J)** GSH levels were determined using the GSH assay kit; **(K,L)** GPX4 mRNA expression was determined using qRT-PCR; and **(M,N)** GPX4 protein levels were determined using the Western blot assay. *N* = 3; *p < 0.05, **p < 0.01, and ***p < 0.001.

To determine whether lncRNA-PRLB regulates ferroptosis in ovarian cancer cells, multiple ferroptosis-related biochemical markers were evaluated following lncRNA-PRLB silencing. ROS fluorescence assays revealed that the knockdown of lncRNA-PRLB markedly increased intracellular ROS levels in both CAOV3 (Figure 3A) and SKOV3 (Figure 3B) cells. Consistently, lipid peroxidation was elevated, as shown by significantly higher MDA levels in CAOV3 (Figure 3C) and SKOV3 (Figure 3D) cells transfected with lncRNA-PRLB siRNA.

Ferroptosis-associated iron accumulation was also enhanced upon lncRNA-PRLB knockdown. Fe^2+^ content was significantly increased in CAOV3 (Figure 3E) and SKOV3 (Figure 3F) cells following siRNA transfection. In parallel, LDH release, an indicator of membrane damage, was significantly elevated in both cell lines (Figures 3G,H), suggesting increased ferroptosis-associated cytotoxicity.

The antioxidant defense system was also impaired by lncRNA-PRLB silencing. GSH levels were significantly reduced in CAOV3 (Figure 3I) and SKOV3 (Figure 3J) cells. Furthermore, GPX4, a key ferroptosis-suppressing enzyme, was markedly downregulated at mRNA (Figures 3K,L) and protein levels (Figures 3M,N) in CAOV3 and SKOV3 cells transfected with lncRNA-PRLB siRNA.

### GPX4 overexpression or ferrostatin-1 treatment reverses the effects of lncRNA-PRLB knockdown on ovarian cancer cell growth, apoptosis, invasion, migration, and chemosensitivity

**Figure 4:**
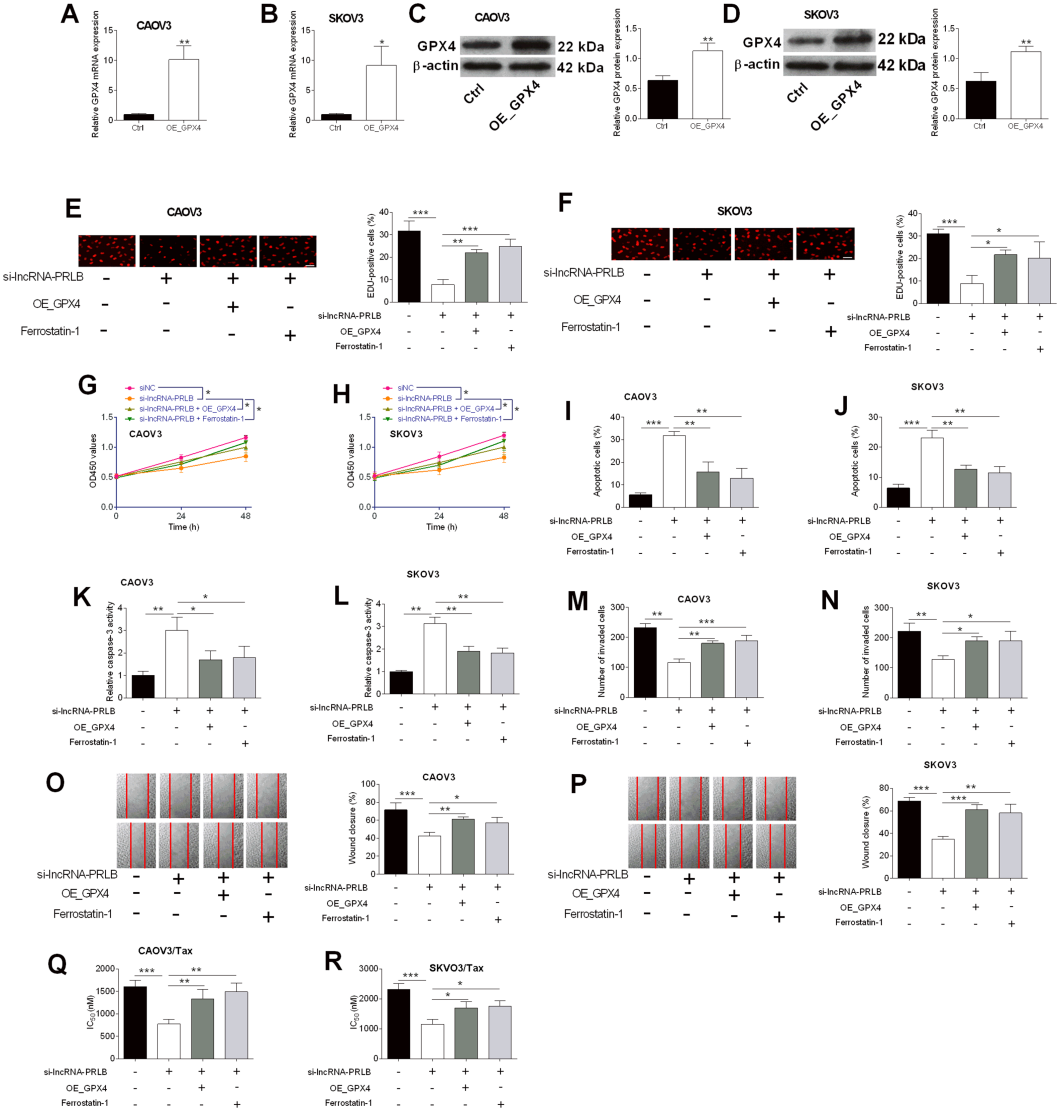
GPX4 overexpression and ferrostatin-1 attenuated the effects of lncRNA-PRLB knockdown on ovarian cancer cell progression and paclitaxel resistance. CAOV3 and SKOV3 cells were transfected with an empty plasmid (Ctrl group) or a GPX4-overexpressing vector (OE_GPX4 group); 48 h after transfection, **(A,B)** GPX4 mRNA expression was determined using qRT-PCR; **(C,D)** GPX4 protein expression was determined using the western blot assay. **(E–P)** CAOV3 and SKOV3 cells were co-transfected with si-lncRNA-PRLB and OE_GPX4 or co-treated with ferrostatin-1 followed by si-lncRNA-PRLB transfection for 48 h; **(E,F)** cell proliferation was evaluated using the EdU incorporation assay; **(G,H)** cell viability was determined using the CCK-8 assay; **(I,J)** cell apoptosis was measured using the TUNEL assay; **(K,L)** caspase-3 activity was determined using the caspase-3 activity assay; **(M,N)** cell invasion was assessed using the transwell invasion assay; and **(O,P)** cell migration was determined using the wound healing assay. **(Q,R)** CAOV3/Tax and SKOV3/Tax cells were co-transfected with si-lncRNA-PRLB and OE_GPX4 or co-treated with ferrostatin-1 followed by si-lncRNA-PRLB transfection for 48 h; the IC50 values of paclitaxel were determined using the CCK-8 assay. *N* = 3; *p < 0.05, **p < 0.01, and ***p < 0.001.

To determine whether GPX4 mediates the regulatory effects of lncRNA-PRLB on ovarian cancer cells, GPX4-overexpressing plasmids were introduced into CAOV3 and SKOV3 cells. qRT-PCR analysis confirmed that GPX4 mRNA levels were elevated in CAOV3 (Figure 4A) and SKOV3 (Figure 4B) cells following GPX4 overexpression. Western blot analysis further verified the corresponding upregulation of GPX4 protein in both cell lines (Figures 4C,D).

We next examined whether GPX4 overexpression or the ferroptosis inhibitor ferrostatin-1 could rescue the biological effects induced by lncRNA-PRLB knockdown. EdU assays revealed that either GPX4 overexpression or ferrostatin-1 treatment markedly attenuated the si-lncRNA-PRLB-induced reduction in cell proliferation in CAOV3 (Figure 4E) and SKOV3 (Figure 4F) cells. Consistent with this observation, CCK-8 assays showed that loss of cell viability caused by lncRNA-PRLB knockdown was significantly reversed by GPX4 overexpression or ferrostatin-1 in both CAOV3 (Figure 4G) and SKOV3 (Figure 4H) cells.

Apoptosis-related phenotypes were also reversed. TUNEL assays revealed that the increase in apoptosis caused by si-lncRNA-PRLB was suppressed by GPX4 overexpression or ferrostatin-1 in both CAOV3 (Figure 4I) and SKOV3 (Figure 4J) cells. Similarly, caspase-3 activity, which was elevated following lncRNA-PRLB knockdown, was significantly reduced upon GPX4 overexpression or ferrostatin-1 treatment in CAOV3 (Figure 4K) and SKOV3 (Figure 4L) cells.

In addition, GPX4 overexpression or ferrostatin-1 treatment effectively counteracted the inhibitory effects of lncRNA-PRLB knockdown on metastatic behaviors. Transwell assays revealed that the decreased invasive capacity induced by si-lncRNA-PRLB was reversed in CAOV3 (Figure 4M) and SKOV3 (Figure 4N) cells. Wound healing assays further showed that impaired migration resulting from lncRNA-PRLB knockdown was restored by GPX4 overexpression or ferrostatin-1 in both CAOV3 (Figure 4O) and SKOV3 (Figure 4P) cells.

Finally, chemoresistance assays revealed that GPX4 restoration mitigated the sensitizing effects of lncRNA-PRLB knockdown on paclitaxel resistance. The reduction in IC₅₀ values of paclitaxel caused by lncRNA-PRLB knockdown was significantly attenuated by GPX4 overexpression or ferrostatin-1 in both CAOV3/Tax (Figure 4Q) and SKOV3/Tax (Figure 4R) cells, indicating that the suppression of ferroptosis is critical for maintaining paclitaxel resistance in ovarian cancer cells.

### GPX4 overexpression or ferrostatin-1 treatment reverses lncRNA-PRLB knockdown-induced ferroptosis in ovarian cancer cells

**Figure 5:**
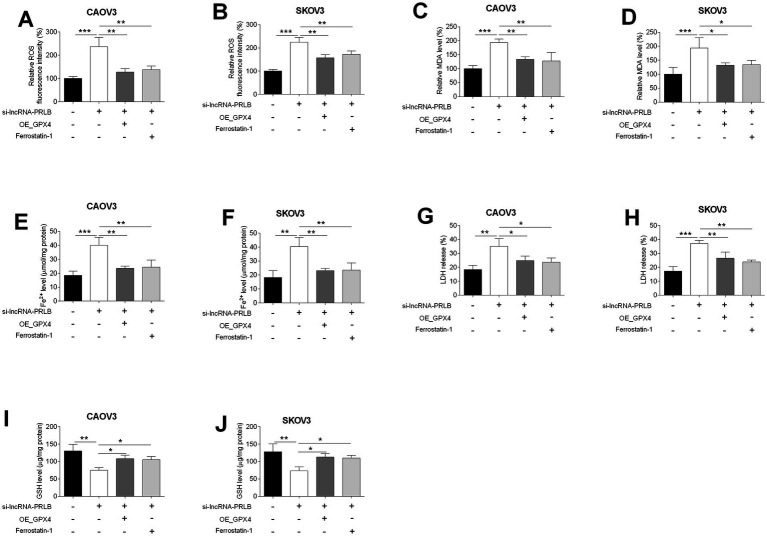
GPX4 overexpression and ferrostatin-1 attenuated the effects of lncRNA-PRLB knockdown on the ferroptosis of ovarian cancer cells. CAOV3 and SKOV3 cells were transfected with empty plasmid (Ctrl group) or GPX4-overexpressing vector (OE_GPX4 group); 48 h after transfection, **(A,B)** ROS levels were determined using the ROS fluorescence detection kit; **(C,D)** MDA levels were measured using the MDA kit; **(E,F)** Fe^2+^ levels in cells were measured using the Fe^2+^ content assay kit; **(G,H)** LDH levels were measured using the LDH release assay; and **(I,J)** GSH levels were determined using the GSH assay kit. *N* = 3; *p < 0.05, **p < 0.01, and ***p < 0.001.

To further clarify whether GPX4 mediates the ferroptotic effects triggered by lncRNA-PRLB knockdown, ferroptosis-associated biochemical markers were examined following co-treatment with GPX4 overexpression or the ferroptosis inhibitor ferrostatin-1. ROS fluorescence assays showed that the elevated ROS levels induced by lncRNA-PRLB siRNA were markedly reduced in both CAOV3 (Figure 5A) and SKOV3 (Figure 5B) cells upon GPX4 overexpression or ferrostatin-1 treatment.

Similarly, the si-lncRNA-PRLB-induced increase in lipid peroxidation, indicated by elevated MDA levels, was significantly attenuated by GPX4 overexpression or ferrostatin-1 in CAOV3 (Figure 5C) and SKOV3 (Figure 5D) cells. Iron accumulation, another hallmark of ferroptosis, was also reversed; Fe^2+^ content was markedly lower in both CAOV3 (Figure 5E) and SKOV3 (Figure 5F) cells following GPX4 overexpression or ferrostatin-1 treatment compared with cells transfected with si-lncRNA-PRLB alone.

Furthermore, LDH release, which was elevated during ferroptosis-associated membrane damage, was significantly reduced in CAOV3 (Figure 5G) and SKOV3 (Figure 5H) cells upon GPX4 overexpression or ferrostatin-1 treatment. In line with these findings, the decrease in intracellular GSH levels induced by lncRNA-PRLB knockdown was effectively rescued in both CAOV3 (Figure 5I) and SKOV3 (Figure 5J) cells.

### LncRNA-PRLB interacts with FUS and regulates GPX4 mRNA stability through a FUS-dependent mechanism

**Figure 6:**
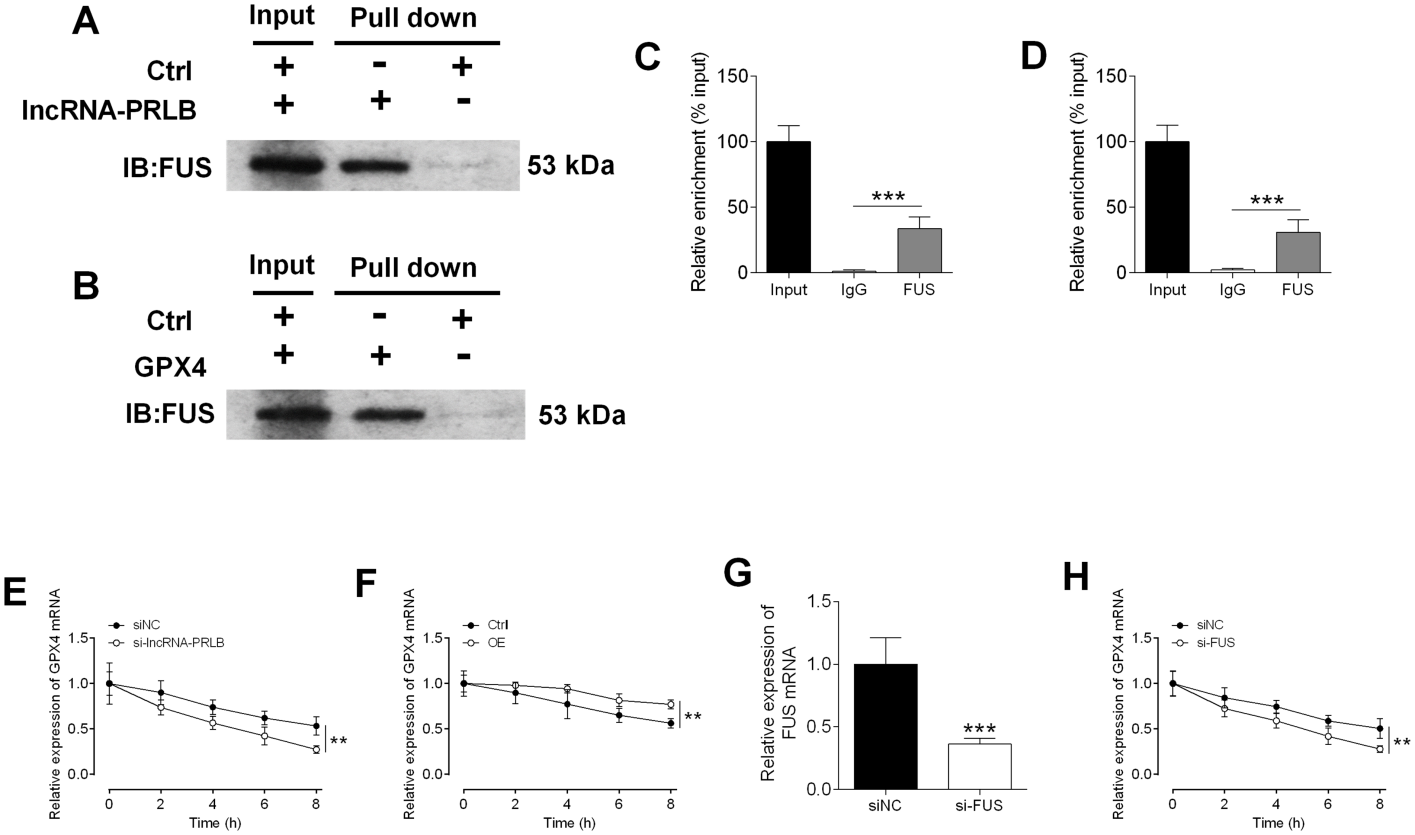
Stability of GPX4 mRNA regulated by lncRNA-PRLB and FUS1. **(A)** The FUS was pulled down by lncRNA-PLBR in CAOV3 cells. **(B)** The FUS was pulled down by GPX4 mRNA in CAOV3 cells. **(C)** The relative enrichment of lncRNA-PRLB by FUS in CAOV3 cells. **(D)** The relative enrichment of GPX4 by FUS in CAOV3 cells. **(E)** The relative expression of GPX4 at different time points of 10 μg/mL actinomycin D treatment after lncRNA-PRLB knockdown in COAV3 cells. **(F)** The relative expression of GPX4 at different time points of 10 μg/mL actinomycin D treatment after lncRNA-PRLB overexpression in COAV3 cells. **(G)** The relative expression of FUS mRNA after FUS siRNA transfection in CAOV3 cells. **(H)** The relative expression of GPX4 at different time points of 10 μg/mL actinomycin D treatment after FUS knockdown in COAV3 cells. *N* = 3; *p < 0.05, **p < 0.01, and ***p < 0.001.

To determine how lncRNA-PRLB regulates GPX4 expression, RNA pull-down and RIP assays were performed to examine potential interactions between lncRNA-PRLB, FUS, and GPX4 mRNA. RNA pull-down assays revealed that FUS protein was efficiently pulled down by lncRNA-PRLB in CAOV3 cells (Figure 6A). Similarly, GPX4 mRNA was able to pull down FUS protein (Figure 6B), indicating that both lncRNA-PRLB and GPX4 mRNA are physically associated with FUS.

RIP assays further confirmed these binding interactions. FUS exhibited strong enrichment of lncRNA-PRLB (Figure 6C) and GPX4 mRNA (Figure 6D), suggesting that FUS interacts with both transcripts in ovarian cancer cells.

To determine whether lncRNA-PRLB regulates GPX4 mRNA stability, actinomycin D degradation assays were performed. Knockdown of lncRNA-PRLB significantly accelerated the degradation of GPX4 mRNA in CAOV3 cells following actinomycin D treatment (Figure 6E), whereas the overexpression of lncRNA-PRLB markedly attenuated the actinomycin D-induced decline in GPX4 mRNA levels (Figure 6F). These results indicate that lncRNA-PRLB stabilizes GPX4 mRNA.

Since both lncRNA-PRLB and GPX4 mRNA bind to FUS, we next assessed whether FUS is required for their stabilizing interaction. FUS siRNA successfully downregulated the expression of FUS in CAOV3 cells (Figure 6G). Importantly, FUS knockdown significantly enhanced the actinomycin D-induced loss of GPX4 mRNA (Figure 6H), mimicking the effect of lncRNA-PRLB knockdown.

### FUS knockdown reverses the oncogenic and anti-ferroptotic effects of lncRNA-PRLB overexpression in ovarian cancer cells

**Figure 7:**
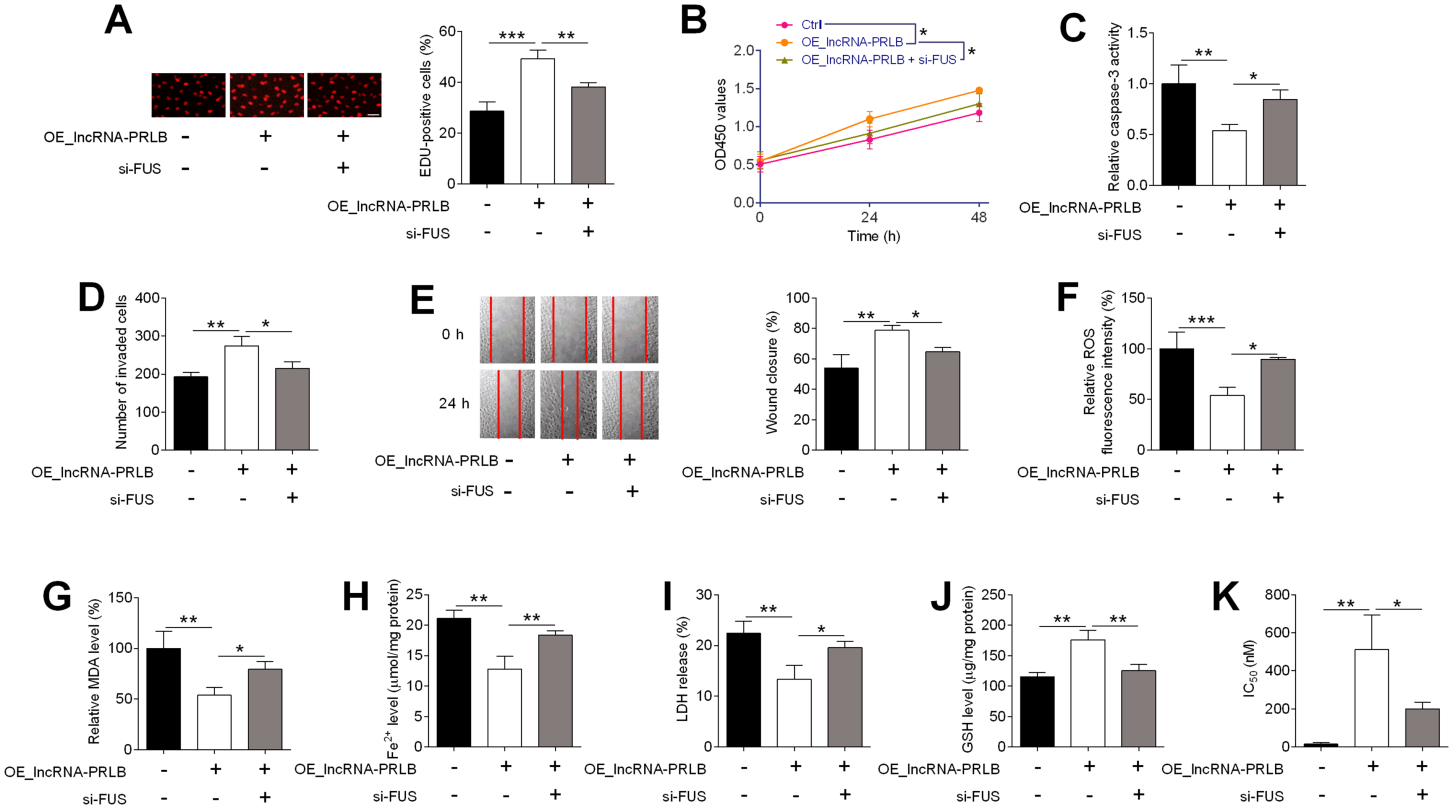
FUS knockdown attenuated the effects of lncRNA-PRLB knockdown on ovarian cancer cell progression, ferroptosis, and paclitaxel resistance. CAOV3 cells were co-transfected with lncRNA-PRLB-overexpressing plasmid and si-FUS for 48 h; **(A)** Cell proliferation was evaluated using the EdU incorporation assay; **(B)** Cell viability was determined using the CCK-8 assay; **(C)** Caspase-3 activity was determined using the caspase-3 activity assay; **(D)** Cell invasion was assessed using the transwell invasion assay; **(E)** Cell migration was determined using the wound healing assay; **(F)** ROS levels were determined using the ROS fluorescence detection kit; **(G)** MDA levels were measured using the MDA kit; **(H)** Fe^2+^ levels in cells were measured using the Fe^2+^ content assay kit; **(I)** LDH levels were measured using the LDH release assay; **(J)** GSH levels were determined using the GSH assay; and **(K)** IC50 values of paclitaxel were determined using the CCK-8 assay. *N* = 3; *p < 0.05, **p < 0.01, and ***p < 0.001.

To evaluate whether FUS is required for the functional effects mediated by lncRNA-PRLB, CAOV3 cells were co-transfected with lncRNA-PRLB-overexpressing plasmids and FUS siRNA. EdU assays showed that FUS knockdown significantly attenuated the enhanced proliferation induced by lncRNA-PRLB overexpression (Figure 7A). Likewise, CCK-8 assays revealed that the lncRNA-PRLB-induced increase in cell viability was markedly reduced following FUS depletion (Figure 7B).

The anti-apoptotic effects of lncRNA-PRLB were also dependent on FUS. Caspase-3 activity, which was decreased by lncRNA-PRLB overexpression, was restored toward basal levels upon FUS knockdown (Figure 7C). Additionally, Transwell invasion assays revealed that the increased invasive capacity driven by lncRNA-PRLB overexpression was significantly diminished by FUS siRNA treatment (Figure 7D). Similarly, wound healing assays showed that FUS knockdown blunted the pro-migratory effects of lncRNA-PRLB in CAOV3 cells (Figure 7E).

Next, ferroptosis-related biochemical parameters were assessed. ROS fluorescence assays showed that FUS knockdown reversed the reduction in ROS levels induced by lncRNA-PRLB overexpression (Figure 7F). Consistently, MDA levels (Figure 7G), intracellular Fe^2+^ content (Figure 7H), and LDH release (Figure 7I), which were all suppressed by lncRNA-PRLB overexpression, were significantly restored by FUS silencing. Conversely, the elevated intracellular GSH levels associated with lncRNA-PRLB overexpression were markedly reduced following FUS knockdown (Figure 7J).

Finally, chemoresistance assays showed that the increase in IC₅₀ values of paclitaxel induced by lncRNA-PRLB overexpression was attenuated by FUS knockdown (Figure 7K), indicating that FUS is required for the lncRNA-PRLB-mediated enhancement of paclitaxel resistance.

## Discussion

In this study, we identify lncRNA-PRLB as a previously uncharacterized regulator of ovarian cancer progression and chemoresistance and uncover a mechanistic axis in which lncRNA-PRLB engages the RNA-binding protein FUS to stabilize GPX4 mRNA and suppress ferroptosis. The observation that lncRNA-PRLB depletion triggers ferroptotic lipid peroxidation, disrupts redox homeostasis, and sensitizes cells to paclitaxel further underscores the centrality of ferroptosis escape in sustaining ovarian cancer cell survival under chemotherapeutic stress. These results align with earlier reports demonstrating that the related transcript PRLB enhances paclitaxel resistance in CAOV3 and SKOV3 ovarian cancer cells by activating RSF1/NF-κB signaling and reducing apoptosis. Additionally, PRLB overexpression in breast cancer promotes proliferation, metastasis, and chemoresistance via miRNA-dependent pathways, including miR-150-5p/remodeling and spacing factor 1 and miR-4766-5p/sirtuin 1 axes (14, 26).

Recent studies have shown that certain therapeutic agents can overcome chemoresistance in part by reactivating ferroptotic programs. For example, the synergistic shikonin–cisplatin combination downregulates GPX4 through heme oxygenase-1-mediated Fe^2+^ accumulation, whereas agrimonolide induces ferroptosis and apoptosis by suppressing stearoyl-CoA desaturase 1 and reducing GPX4 and SLC7A11 levels (27, 28). Transcriptional regulators such as PAX8 sustain GPX4-dependent ferroptosis resistance by upregulating GCLC (29), while oncogenic signaling factors such as RNLS promote ovarian cancer growth by activating STAT3–PI3K/AKT signaling and maintaining high GPX4 and GSH abundance (30). Moreover, GPX4 inhibition sensitizes these cells to paclitaxel, further highlighting GPX4 as a central survival node in chemotolerant states (31). Our data reveal a distinct layer of regulation in which lncRNA-PRLB scaffolds the RNA-binding protein FUS to enhance GPX4 mRNA stability. This indicates that ferroptosis sensitivity can be modulated not only by metabolic flux, lipid peroxidation pathways, or antioxidant systems but also by RNA-binding events that govern transcript turnover.

Accumulating evidence shows that FUS acts as a central regulatory hub co-opted by diverse oncogenic non-coding RNAs to stabilize pro-tumorigenic transcripts. In breast cancer, LINC00460 recruits FUS to promote MYC mRNA maturation and drives metastasis and doxorubicin resistance (32); in prostate cancer, the lncRNA EMX2OS binds FUS to synergistically activate the cGMP–PKG pathway and suppress tumor growth (33); in cervical cancer, ABHD11-AS1 prevents FUS-mediated degradation of ABHD11 mRNA, thereby sustaining EGFR signaling (34); in nasopharyngeal carcinoma, FAM225A stabilizes CENP-N via FUS to activate cGAS–STING signaling (35); and in pancreatic cancer, the tumor-suppressive ST18-AS1 anchors FUS in the cytoplasm to preserve ST18 mRNA stability (36). In ovarian cancer specifically, two recent studies further highlight the breadth of FUS-mediated regulatory networks: FUS stabilizes USP7 mRNA to promote bevacizumab resistance through PTK2 deubiquitination, and the circANKRD17/FUS/FOXR2 axis enhances paclitaxel resistance by preventing FOXR2 degradation (18, 37). We showed that FUS directly binds both lncRNA-PRLB and GPX4 mRNA, forming a functional ribonucleoprotein complex essential for maintaining GPX4 expression and preventing ferroptosis. Notably, FUS knockdown phenocopied the effects of lncRNA-PRLB depletion. Loss of FUS abolished the oncogenic and ferroptosis-suppressive activities of lncRNA-PRLB overexpression. These findings support a model in which lncRNA-PRLB exerts its biological function primarily through FUS, positioning FUS as a critical determinant in the maintenance of ferroptosis resistance in ovarian cancer.

The discovery that manipulating the lncRNA-PRLB/FUS/GPX4 axis alters paclitaxel sensitivity provides a link between ferroptosis regulation and chemotherapy response (20). Previous studies have suggested that chemoresistant ovarian cancer cells adopt antioxidant and iron-buffering strategies to evade ferroptosis (38–40), but the upstream drivers of this phenotype have remained unclear. Our results revealed that lncRNA-PRLB maintains a ferroptosis-resistant state conducive to drug tolerance, while its suppression reactivates ferroptotic vulnerability and restores paclitaxel responsiveness. These data support the concept that ferroptosis induction represents a therapeutically tractable vulnerability in chemoresistant ovarian cancer.

This study has several limitations. First, the extent to which lncRNA-PRLB contributes to ferroptosis resistance *in vivo* remains to be established. Future studies incorporating patient-derived xenografts or genetically engineered mouse models will be essential to validate its role in tumor maintenance and therapy response. Second, although our RNA pull-down, RIP, and actinomycin D assays support a functional lncRNA-PRLB–FUS–GPX4 axis, we did not define the structural or domain-specific determinants of these interactions; therefore, the current study cannot establish precise binding interfaces. Future studies using FUS truncation/domain-mutant constructs combined with RNA immunoprecipitation/cross-linking and immunoprecipitation approaches and lncRNA-PRLB deletion mapping will be important to pinpoint the interaction domains and to determine whether lncRNA-PRLB functions as a scaffold to enhance FUS occupancy on GPX4 mRNA. Third, given that FUS regulates a broad repertoire of transcripts, it is plausible that additional mRNAs involved in metabolism, redox homeostasis, or cell survival participate in the PRLB–FUS network; transcriptome-wide RNA immunoprecipitation sequencing or cross-linking and immunoprecipitation followed by sequencing profiling could illuminate the full scope of this regulatory landscape. Finally, while our findings focus on paclitaxel, ferroptosis suppression is implicated in resistance to multiple chemotherapeutic agents, raising the possibility that lncRNA-PRLB contributes to multidrug resistance in ovarian cancer. Fourth, although ferrostatin-1 rescue experiments support that the phenotypes induced by lncRNA-PRLB silencing are primarily ferroptosis-dependent, our study did not comprehensively dissect potential crosstalk between ferroptosis and apoptosis. In particular, we did not perform systematic pathway separation experiments (e.g., pan-caspase inhibition, genetic perturbation of core apoptotic regulators, or time-resolved profiling of ferroptotic versus apoptotic markers) to determine whether the observed apoptotic features represent secondary downstream signaling or a partially parallel death program. Fifth, our conclusions are based on CAOV3/SKOV3 and their paclitaxel-resistant derivatives; validating the lncRNA-PRLB/FUS/GPX4 axis in additional ovarian cancer models (e.g., A2780 or OVCAR3) will strengthen generalizability.

## Conclusion

In summary, our study identifies lncRNA-PRLB as a key regulator of ferroptosis and chemoresistance in ovarian cancer and reveals a mechanistic axis in which lncRNA-PRLB recruits FUS to stabilize GPX4 mRNA. These findings advance our understanding of ferroptosis regulation at the RNA level, uncover a previously unrecognized lncRNA–RBP interaction that supports tumor fitness, and suggest new therapeutic opportunities for targeting ferroptosis resistance in ovarian cancer. By situating ferroptosis within the broader framework of RNA-based gene regulation, this study underscores the potential of lncRNAs and their RBP partners as actionable nodes in overcoming treatment resistance.

## Data Availability

The original contributions presented in the study are included in the article/Supplementary material, further inquiries can be directed to the corresponding author.
