# Supplementary figures and images for: LncRNA-PRLB drives ovarian cancer progression and chemoresistance by stabilizing GPX4 mRNA through the FUS-mediated suppression of ferroptosis
Source: Front Med (Lausanne). 2026 Feb 17;13:1759058. doi: 10.3389/fmed.2026.1759058 (PMC12953528; doi:10.3389/fmed.2026.1759058)

Figure 3A

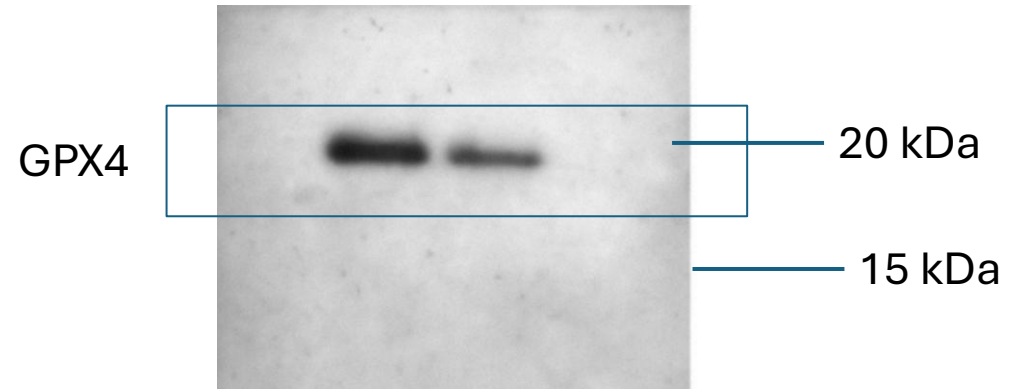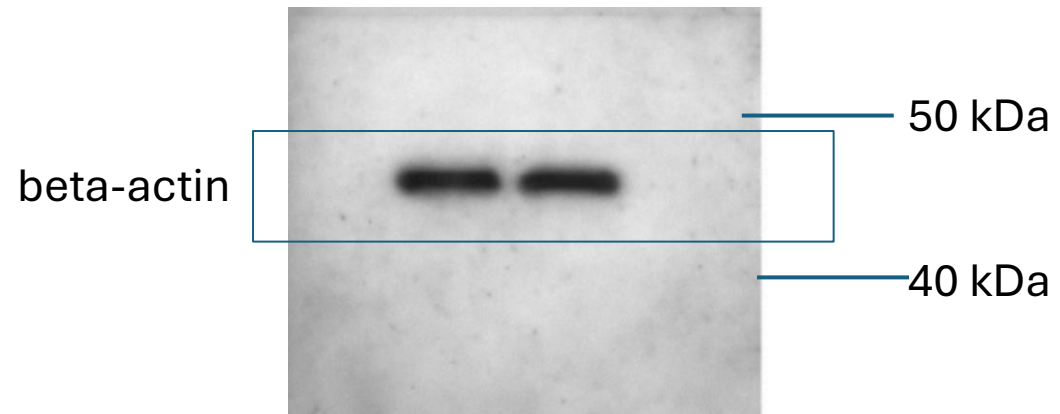

Figure 3B

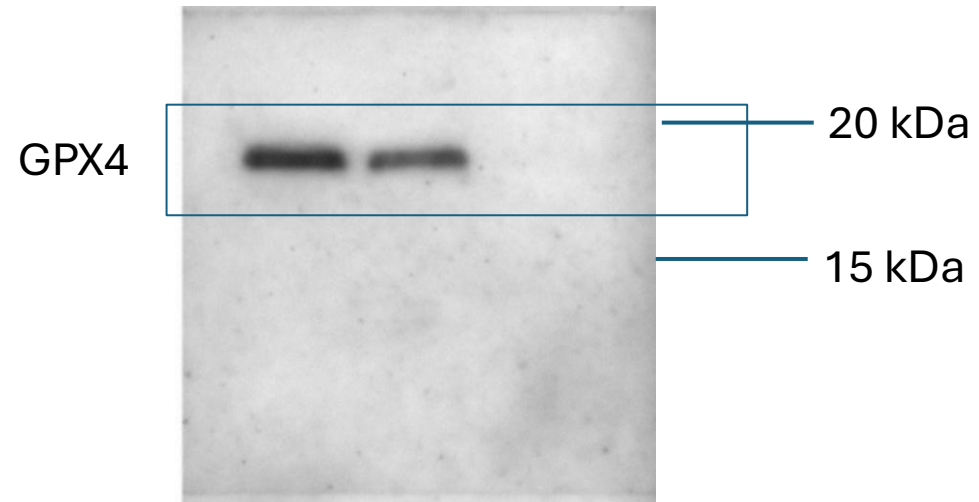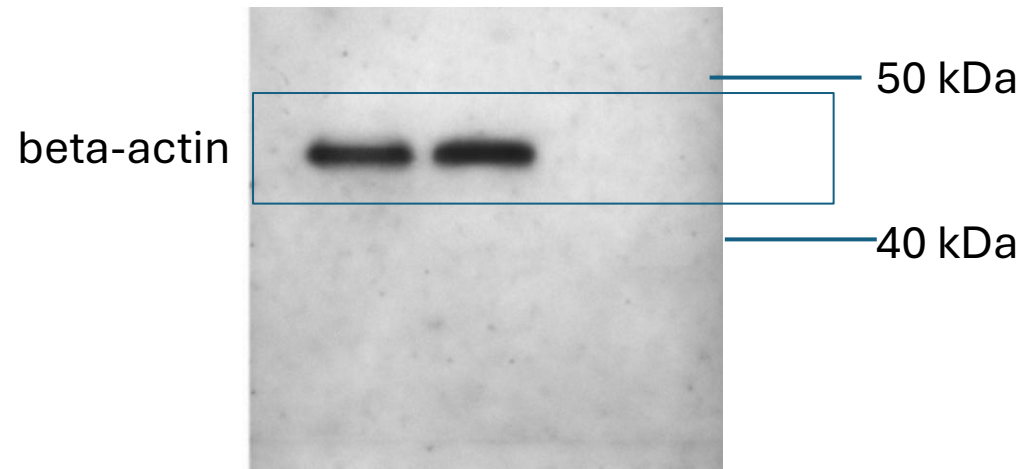

Figure 4C

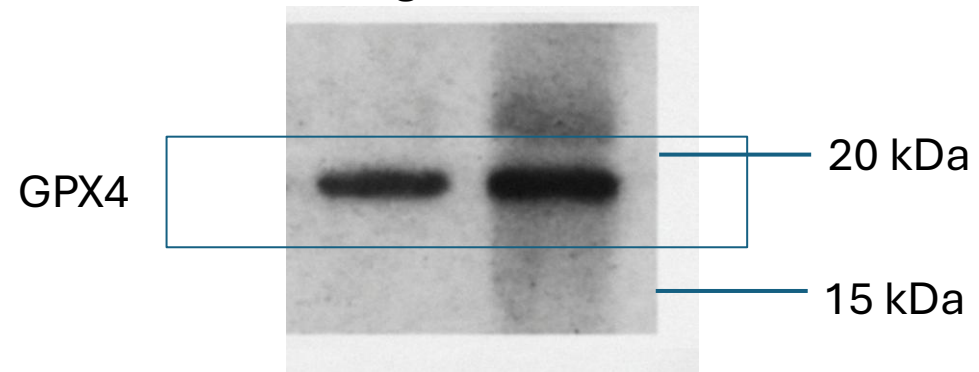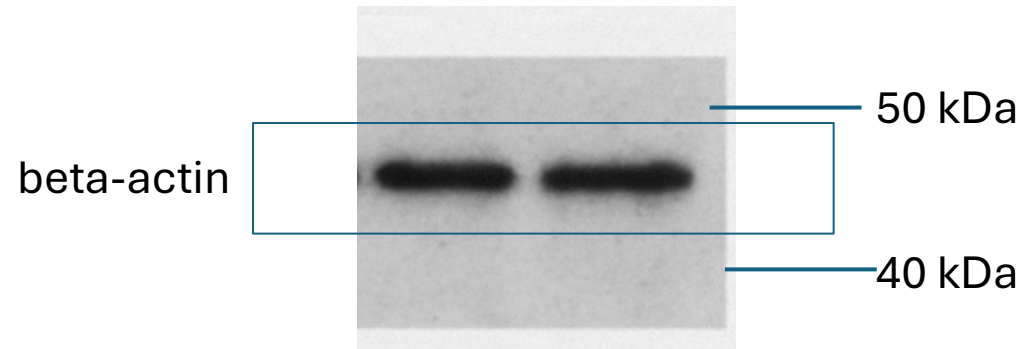

Figure 4D

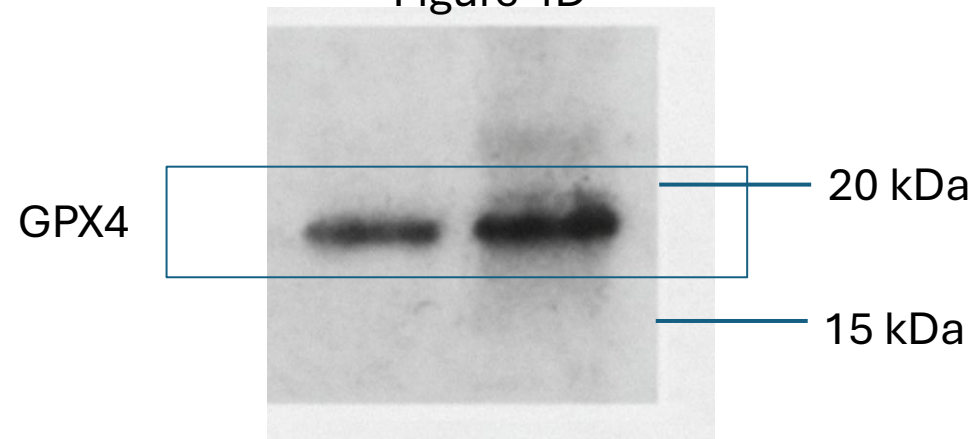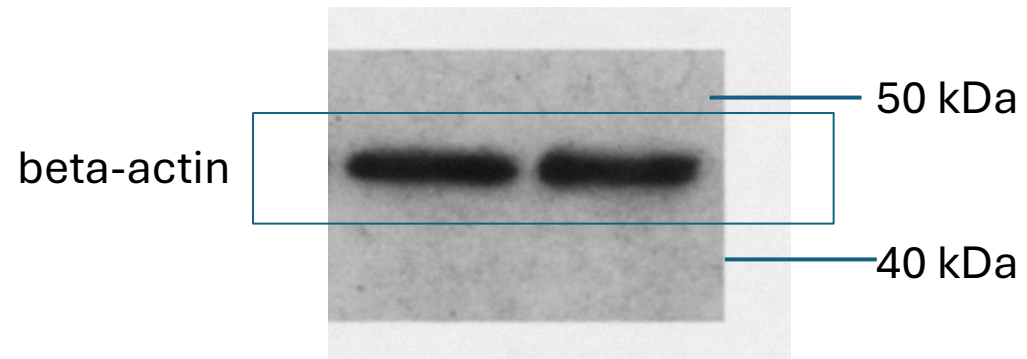

Figure 6A

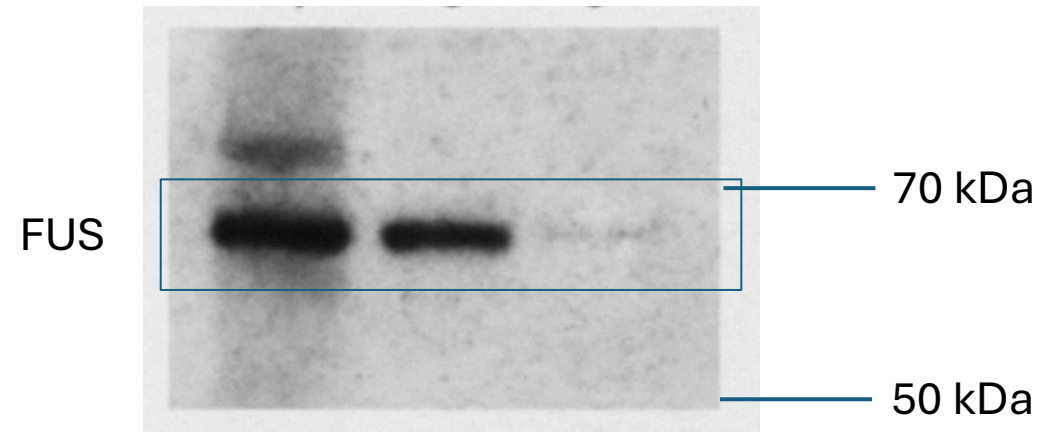

Figure 6B

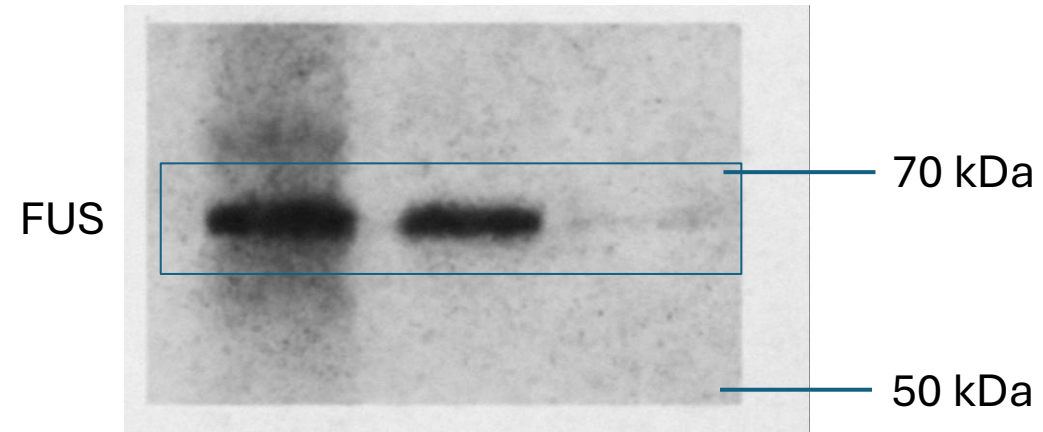

Supplement: Supplementary file 1 [file Data_Sheet_1.pdf]
