# Supplementary material for: LncRNA-PRLB drives ovarian cancer progression and chemoresistance by stabilizing GPX4 mRNA through the FUS-mediated suppression of ferroptosis
Source: Front Med (Lausanne). 2026 Feb 17;13:1759058. doi: 10.3389/fmed.2026.1759058 (PMC12953528; doi:10.3389/fmed.2026.1759058)
Supplement: Supplementary file 2 [file Table_1.docx]

Table S1. SiRNA sequences

|  | Sequences |
| --- | --- |
| SiRNA for lncRNA-PRLB | 5’-AGAGAGUGACUGCUUCUCA-3’ |
| Scrambled siRNA for lcnRNA-PRLB | 5’-UGAGUACGUGAACUCUAGC-3’ |
| SiRNA for FUS | 5’-GCCAAGAUCAAUCCUCCAU-3’ |
| Scrambled siRNA for FUS | 5’-UUCUCCGAACGUGUCACGU-3’ |
